# *Sdt97*: A Point Mutation in the 5′ Untranslated Region Confers Semidwarfism in Rice

**DOI:** 10.1534/g3.116.028720

**Published:** 2016-04-28

**Authors:** Jiping Tong, Zhengshu Han, Aonan Han, Xuejun Liu, Shiyong Zhang, Binying Fu, Jun Hu, Jingping Su, Shaoqing Li, Shengjun Wang, Yingguo Zhu

**Affiliations:** * Department of Rice Breeding, Tianjin Crop Institute, Tianjin P.R., 300384 China; † Department of Rice Breeding, Shangdong Rice Institute, Shandong Province, P.R. 250100 China; ‡ Department of Crop Molecular Biology, Institute of Crop Sciences of CAAS, Beijing P.R., 10081 China; § Key Laboratory of MOE for Plant Developmental Biology, College of Life Sciences, Wuhan University, Wuhan, Hubei Province P.R., 430072 China

**Keywords:** semidwarfism, *Sdt97*, transversion, map-based cloning, qRT-PCR, complement test, rice (*Oryza sativa* L.)

## Abstract

Semidwarfism is an important agronomic trait in rice breeding programs. The semidwarf mutant gene *Sdt97* was previously described. However, the molecular mechanism underlying the mutant is yet to be elucidated. In this study, we identified the mutant gene by a map-based cloning method. Using a residual heterozygous line (RHL) population, *Sdt97* was mapped to the long arm of chromosome 6 in the interval of nearly 60 kb between STS marker N6 and SNP marker N16 within the PAC clone P0453H04. Sequencing of the candidate genes in the target region revealed that a base transversion from G to C occurred in the 5′ untranslated region of *Sdt97*. qRT-PCR results confirmed that the transversion induced an obvious change in the expression pattern of *Sdt97* at different growth and developmental stages. Plants transgenic for *Sdt97* resulted in the restoration of semidwarfism of the mutant phenotype, or displayed a greater dwarf phenotype than the mutant. Our results indicate that a point mutation in the 5′ untranslated region of *Sdt97* confers semidwarfism in rice. Functional analysis of *Sdt97* will open a new field of study for rice semidwarfism, and also expand our knowledge of the molecular mechanism of semidwarfism in rice.

Dwarfism is one of the most important agronomic traits of rice. The introduction of high-yielding dwarf cultivars, together with the application of large amounts of fertilizer and pesticides—termed the ‘Green Revolution’—has contributed substantially to the significant increase in rice production, and largely averted the chronic food shortage that was feared after the rapid expansion of the world population in the 1960s (Khush 2001; Sasaki *et al.* 2002; Hedden 2003; Muangprom and Osborn 2004).

Utilization of dwarf rice cultivars was one of the greatest achievements in rice breeding in the 20th century. Before the introduction of the dwarf gene to rice improvement programs, rice cultivars were all tall; the stems of tall rice plants were not strong enough to support the heavy grain of the high-yielding varieties, and this resulted in plants falling over. This process, known as lodging, resulted in large yield losses (Hedden 2003).

Semidwarf plants possess short, strong stalks, exhibit less lodging, and a greater proportion of assimilation partitioned into the grain, resulting in further yield increases (Hedden 2003). Reducing plant height to breed high-yielding, nonlodging, rice varieties has become a predominant strategy for breeders since the 1960s (Hargrove and Cabanilla 1979). Dwarf genes have been utilized extensively in plant breeding to improve lodging resistance (Khush 2001; Sasaki *et al.* 2002).

The dwarfing gene originated from a Chinese cultivar, Dee-geo-woo-gen, which was used in a breeding program in Taiwan during the 1950s to produce the highly successful Taichung Native 1. This cultivar was also used later at the International Rice Research Institute (IRRI) in the Philippines to produce IR-8. Semidwarf, high-yielding cultivars have also been produced independently in the People’s Republic of China, Japan and the USA. The semidwarf trait can be attributed to the different alleles of a single recessive gene, *sd1*, even where the parent strains have been selected or produced independently (Hedden 2003; Sasaki *et al.* 2002; Kikuchi and Futsuhara 1997).

In previous research, we isolated a dominant semidwarf mutant gene *Sdt97* (Tong *et al.* 2007). In this article, we report current progress in the molecular cloning of *Sdt97*.

## Materials and Methods

### Plant materials and growth conditions

Plant materials used in this study are the semidwarf mutant (Y98149), the tall wild type (Y98148), Huajingxian74, an elite *indica* variety, F_7_ residual heterozygous lines (RHL), and F_7:8_ residual heterozygous lines (RHL). Y98149 was isolated originally from an F_6_ generation nursery selection of a medium *japonica* rice cross between M9056 and R8018 Xuan. Y98149 and Y98148 are near isogenic lines (NIL) (Tong *et al.* 2003).

Field trials were carried out on paddy fields in two locations. (1) During the natural rice growing seasons in Hefei in 2006, Y98149, Y98148, Huajingxian74, and F_7_ RHL were planted at the Experimental Station at Hefei (31 × N, 117 × E), Anhui province, China. Seeds were sown in seed beds on April 15–20, 2006. On June 15–20, 1-month old seedlings were transferred to the paddy field for further growth. (2) During the natural early rice growing seasons in Sanya in 2006–2007, Y98149, Y98148, Hua jingxian74, and F_7:8_ RHL were planted in the field at experimental stations at Sanya (18 × N, 109 × E), Hainan province, China. Seeds were sown in seed beds on November 15–20, 2006. On December 15–20, 2006, 1-month old seedlings were transferred to the paddy field for further growth.

The planting density was 13.3 cm between plants in a row, and the rows were 16.7 cm apart. Field management, including irrigation, fertilizer application, and pest control, followed normal agricultural practice.

### Phenotypical characterization and genetic analysis

In this research, plant heights were recorded at maturity in the paddy field. Plant height was measured from ground level to the plant material tassel tip. Statistical significance was assessed using Student’s *t*-test. Probability values (*P*) 0.05 were considered significant. Segregation of plant height phenotype in RHL segregated progenies was analyzed for goodness of fit in the ratio of 3:1 using a Chi-square test (χ^2^ < χ^2^_0.05_, 1 = 3.84). Each quantitative real-time reverse transcription-polymerase chain reaction (qRT-PCR) was performed for three biological replicates. The ΔΔCt method was used for data analysis.

### Mapping population development

An intersubspecific cross between semidwarf mutant Y98149 (japonica cv.) and Hua- jing-xian74 (indica cv.) was developed for the *Sdt97* mapping. A F_6_ recombinant inbred line population (RIL) was identified to be a RHL, and was used in the rough mapping of *Sdt97* (Tuinstra *et al.* 1997; Yamanaka *et al.* 2005; Tong *et al.* 2007).

**Figure 1:**
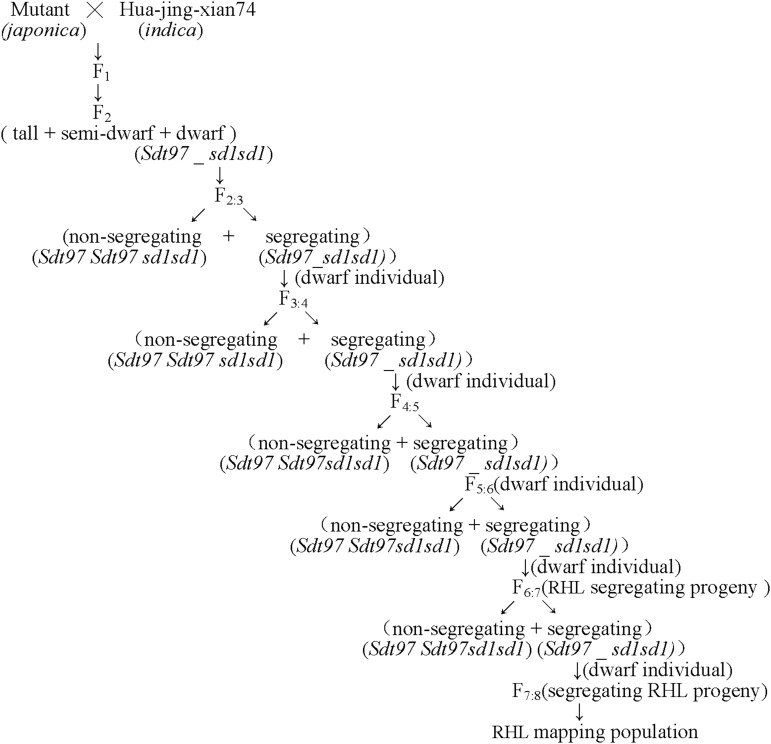
Development of the RHL mapping population.

In the fine mapping study of *Sdt97*, a large scale F_7_ RIL population was constructed. Out of this segregating F_7_ RHL population, F_7_ recessive tall individuals were selected for the fine mapping of *Sdt97*. For the genetic validation, or progeny testing of the recessive F_7_ individuals, F_7:8_ RHL derived from these tall F_7_ recessive individuals were further planted in the field at experiment stations in Sanya (18 × N, 109 × E), Hainan province, China. The developmental process of the RHL population was as shown in Figure 1.

### DNA extraction and molecular marker development

Fresh leaves were collected and ground in liquid nitrogen. DNA was extracted from the ground tissues using the modified CTAB (celytrimethyl ammonium bromide) method (Rogers and Bendich 1998).

Single nucleotide polymorphisms (SNPs) and cleaved amplified polymorphic sequence (CAPS) markers were developed by sequencing the PCR products amplified from *japonica* cv. semidwarf mutant Y98149, and *Indica* cv. Huajingxian74 in the rough mapping region of *Sdt97*. PCR products were cloned into the vector pGEM-T (Promega, USA) for sequencing, and for developing SNPs. PCR amplifications followed the profile: 94° for 3 min, 35 cycles of 94° for 1 min, 55° for 1 min and 72° for 1 min, with a final extension of 72° for 5 min. The markers, which showed polymorphism between Y98149 and Huajingxian74, were then utilized for *Sdt97* fine mapping.

The LightCycler480 Real-Time PCR System was employed for SNP Genotyping. PCR amplification was performed in a 96-well plate in the LightCycler 480 Real-Time PCR System. Reaction volume was 10 μl; 2 μl of genomic DNA (10 ng/μl) was added to 8 μl of the reaction master mixture consisting of 1× LightCycler 480 High Resolution Melting Master (containing the proprietary ds-DNA saturating binding dye), with 2.5 mM MgCl_2_ (Roche Diagnostics, Germany), and 0.5 μM of forward and reverse primers. The PCR program started with an initial denaturation of 10 min at 95°, and continued with 40 cycles of 10 sec at 95°, 15 sec at 60°, and 10 sec at 72°. For high resolution melting (HRM), the plate was heated from 65° to 95°, performing 25 acquisitions per 1° (Norambuena *et al.* 2009).

### Map-based cloning

Using the RHL, *Sdt97* was mapped to a 118-kb region on chromosome 6 flanked by two STS markers, N6 and TX5(Tong *et al.* 2003). Bulked segregant analysis (BSA) (Michelmore *et al.* 1991) combined with recessive class analysis (RCA) (Chen *et al.* 2005; Pan *et al.* 2003; Zhu *et al.* 2004; Zhang *et al.* 1994) were used to identify molecular markers linked to the mutant gene *Sdt97* in the fine mapping of *Sdt97*. Genomic DNA from 30 tall individuals and 30 dwarf individuals in the F_6:7_ segregated progenies were pooled to create the tall and dwarf DNA bulks, respectively. The parental DNA, and the two bulks, were used for BSA. Polymorphic markers from the two parents were screened against the two DNA bulks, and polymorphic markers between the two DNA bulks were screened against the entire population of tall recessive individuals in the F_6:7_ segregated population.

Using the markers developed in this research, the mutant gene *Sdt97* was finally mapped, the candidate genes detected in the target region were PCR-amplified and sequenced for mutation detection. The average length of the PCR products was 1.5 kb, and the PCR products were sequenced directly.

### RNA manipulation and qRT-PCR analysis

To analyze the expression pattern of *Sdt97* carried by the semidwarf mutant Y98149, *sdt97* carried by tall wild-type Y98148, and the gene expression of *Sdt97* in transgenic lines, samples were prepared from Y98149, Y98148, and the transgenic T_1_ lines of F_4_ plants at seedling stage, tillering stage, elongation stage, and milk ripe stage, respectively.

Fresh plant tissues of above-ground rice were harvested, and immediately ground to fine powder in liquid nitrogen. Total RNA was isolated using TRIzol reagent (Invitrogen) according to the manufacturer’s instructions. The TransScript One-Step gDNA Removal and cDNA Synthesis SuperMix (TransGen Biotech) were used to synthesize cDNA using an anchored oligo(dT)_18_ primer according to the kit instructions.

qRT-PCR (20 μl reaction volume) was carried out using 0.5 μl cDNA, 0.2 μl of each gene-specific primer, and SYBR Premix Ex Taq Kit (TaKaRa) in a LightCycler Real Time PCR according to the manufacturer’s instructions. Reaction conditions were 95° for 30 sec, followed by 40 cycles of 95° for 5 sec, and 60° for 31 sec. The standard procedure for melt curve analysis followed the amplification cycles. The rice actin gene was used as the endogenous control for normalization. Each real-time PCR analysis was performed for three biological replicates. The relative quantification method (ΔΔCt) was used for data analysis.

The gene-specific primers used for qRT-PCR are as follows: *Sdt97*-specific Q1 (forward primer: GTCCGGTCGCTGAACGTG; reverse primer: GGCTTCGGCGAGGGCTT), located in the first exon coding section of *Sdt97*; the expected length of the qRT-PCR product was 113 bp. *Sdt97*-specific Q2 (forward primer: CATGTGGAACAGTGATGCGG; reverse primer: AACTGG GTTGCATTACTGACACA), located towards the end of the noncoding cDNA section of *Sdt97*; the expected length of qRT-PCR product was 136 bp. Actin-specific (LOC_Os03g61970) (forward primer: ATCCTTGTATGCTAGCGGTCGA; reverse primer: ATCCAACCGGAGGATAGCATG).

### Vector constructions and plant transformation

To confirm further that the mutated traits were caused by mutant gene *Sdt97*, we constructed transgenic plants for the complement test of *Sdt97*. The Gateway cloning system was adopted, and the Gateway vector pGWB12:[(35S promoter,N-FLAG) (–35S promoter-FLAG-R1-CmR-ccdB- R2–)] (Invitrogen) was used for the *Sdt97* complementarity test. We wanted to test the effect of the point mutation in the 5′ untranslated region (5′-UTR) on the gene expression of *Sdt97* itself. Considering that the point mutation in the 5′-UTR may be associated with the promoter of *Sdt97*, perhaps the point mutation in the 5′-UTR is part of the promoter itself, thus pGWB3: [(no promoter, C-GUS) (-R1- CmR-ccdB-R2-GUS -)] (Invitrogen nomenclature), a no promoter Gateway vector, was also used in the complementary test of *Sdt97*.

Two *Sdt97* genomic DNA fragments were amplified by PCR from the genomic DNA using the following primers: BF_1_: GTGGGGACAAGTTTGTACAAAAAAGCA GGCTTCCCTCTCCTCTCCTCTCACCACC, BR_1_: GTGGGGACCACTTTGTACAAGAAAGCTGGGTCTGTTAACTGATTGAGGAAGATTTTTCA, BF_2_: GTGGGGACAAGTTTGTACAAAAAAGCAGGCTTCGTGAGATAATGCCGGGCCC, and BR_2_: GTGGGGACCACTTTGTACAAGAAAGCTGGGTCAGGTATCCTGCAGTTCTGCATG. A 2701-bp genomic DNA fragment containing the entire *Sdt97*, and a 3116-bp genomic DNA fragment containing the entire *Sdt97*, the 174 bp 5′ upstream sequence and the 243 bp 3′ downstream sequence, were amplified with high fidelity using KOD-Plus-Neo (TOYOBO, Japan). The PCR amplification products were mixed with Donor vectors (Invitrogen nomenclature) and BP Clonase enzyme mix to construct an Entry clone. Then, the Entry clone was transferred into the Gateway vector using the enzyme mix, and LR Clonase to construct the Expression clone. The expression clone was constructed from the 3116-bp PCR product and pGWB12, and was designated as F_4_. The expression clones constructed from the 2701 bp and 3116 bp PCR products and pGWB3 were named T_6_ andT_15_, respectively. The expression clone was introduced into the *Agrobacterium* strain EHA105 by electroporation, and transformation was carried out as described (Hiei *et al.* 1994; Xue *et al.* 2008).

Positive transgenic plants were detected by sequencing the PCR products of the genomic DNA using a pair of primers (forward primer: CCTCTCCTCTCCTCTCACCACC; reverse primer:TAATCCCAGCCCAGGGTTCG) designed according to the sequence mutation at the promoter region. The PCR products were 223 bp, and it was easy to separate the transgenic plants from that of transgenic acceptors line or tall wild type plants. Transgenic lines were selected using hygromycin treatment (15 mg L–1).The transgenic rice plants were transferred to and grown in experimental fields from January to April in Sanya, Hainan Province, and from May to October in Hefei Anhui Province China 2014.

### Data availability

The authors state that all data necessary for confirming the conclusions presented in the article are represented fully within the article.

## Results

### Isolation and phenotypic characterization of the semidwarf mutant

In 1997, a semidwarf mutant was isolated from the tall F_6_ progenies derived from the cross between M9056 and R8018 XUAN (Tong *et al.* 2001). The semidwarf mutant was deduced to result from a spontaneous mutation, and not from the cross-fertilization between the tall wild type and other dwarf rice cultivars (Tong *et al.* 2003). It is the NIL to the tall wild type in the same selected nursery, the semidwarf mutant was named Y98149, and the tall wild type was named Y98148 (Tong *et al.* 2003).

The length of each internode of the mutant is almost uniformly shortened, resulting in an elongation pattern similar to that of the wild-type plant (Table 1). According to the grouping of rice dwarf cultivars proposed by Takahashi, the semidwarf mutant belongs to the dn-type (Takahashi *et al.*and Takeda 1969).

**Table 1:** Plant height, panicle, and elongate internode length of semidwarf mutant and tall wild type

Item	Semidwarf Mutant	Tall Wild Type	Percentage Decrease (%)
Plant height	75.87 ± 4.55	104.63 ± 4.68	−27.49*
Panicle length	27.12 ± 2.44	28.69 ± 2.17	−5.47
Length of the first internode under panicle	17.95 ± 1.67	20.34 ± 1.62	−11.75*
Length of the second internode under panicle	24.21 ± 2.37	30.48 ± 2.72	−20.57*
Length of the third internode under panicle	12.09 ± 1.22	19.44 ± 1.48	−37.81*
Length of the fourth internode under panicle	7.37 ± 0.89	12.10 ± 1.48	−39.09*
Length of the fifth internode under panicle	5.88 ± 1.05	8.98 ± 0.97	−34.52*
Length of the sixth internode under panicle	2.96 ± 1.17	4.82 ± 1.27	−38.59*
Length of the seventh internode under panicle	0.90 ± 1.16	1.61 ± 2.37	−44.10
Length of the eighth internode under panicle	0.21 ± 0.80	0.27 ± 0.75	−22.22
Length of the ninth internode under panicle	0.07 ± 0.29	0.10 ± 0.42	−30.00

* Significance at *P* < 0.01 level; statistical significance was assessed using Student’s t-test.

**Figure 2:**
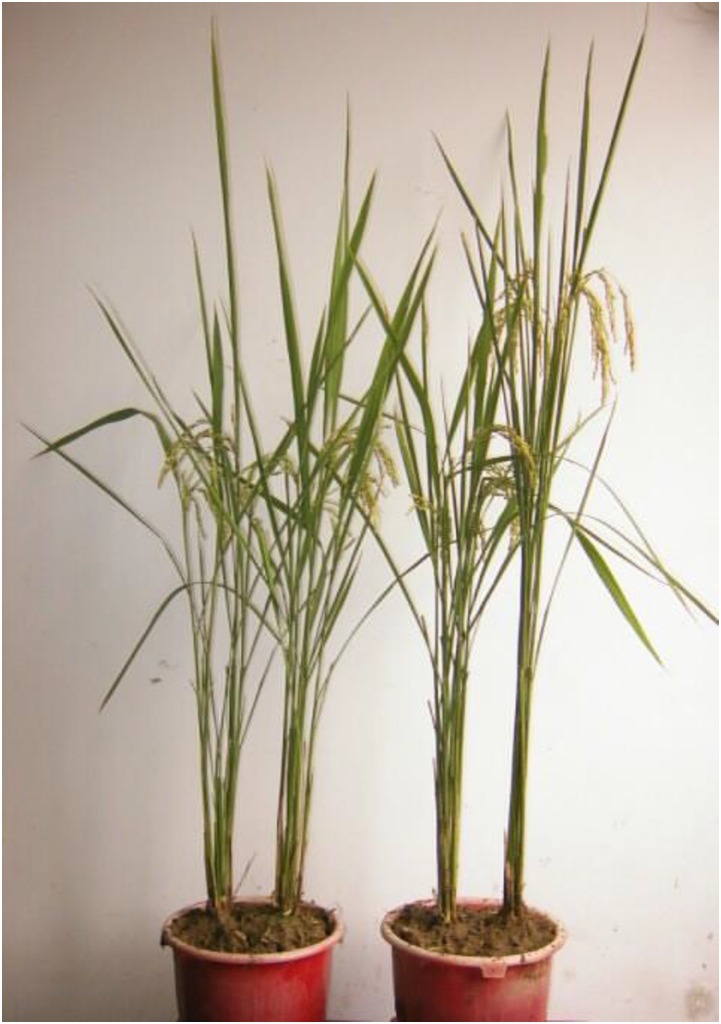
Tall wide type (WT), semidwarf mutant (M), and the F_1_ progeny derived from reciprocal crosses between M and WT [from right to left: WT, M, (WT/M)F_1_, (M/WT)F_1_].

Research into the genetic character of the semidwarf mutant revealed that there was only one dominant gene locus involved in the control of the semidwarfism, and the semidwarfism expression of the mutant was not affected by its cytoplasm (Figure 2). The results of classic genetic allelic tests revealed that the semidwarf mutant gene is a new kind of semidwarf gene reported in rice; the mutant gene was temporarily designated *Sdt97* (Tong *et al.* 2003, *et al.* 2007).

### RHL population development

For the *sdt97* mapping, an intersubspecific cross between semidwarf mutant Y98149 (japonica cv.) and Hua-jing-xian74 (indica cv.) was developed. A F_6_ recombinant inbred line population (RIL), which originated from the intersubspecific cross, was identified as an RHL (Yamanaka *et al.* 2005; Tuinstra *et al.* 1997; Tong *et al.* 2007). The heterozygous chromosomal region is approximately 25.5 cM and 6646 kb, starting from RM3430 and ending at RM6395. This was initiated by different parents, one carrying the *Sdt97* gene derived from the mutant, and the other carrying the *sdt97* gene derived from Hua-jing-xian74 (Table 2). This F_6_ residual heterozygous lines population was used in the rough mapping of *Sdt97*, and the semidwarf mutant gene *Sdt97* was mapped on the long arm of chromosome 6 at the interval between two STS markers N6 and TX5, with a genetic distance of 0.2 cM and 0.8 cM, respectively (Tong *et al.* 2007).

**Table 2:** Constituents of chromosome 6 of the RHL

Marker Name	Gene-Type*^a^*	Location (bp)
RM8019	AA	485930–486040
RM589	AA	1380876–1381023
RM588	AA	1611413–1611510
RM190	AA	1764638–1764760
RM225	AA	3416596–3416728
RM584	AA	3416595–34516764
RM253	aa	5425498–5425631
RM276	aa	6230045–6230185
RM4128	aa	6644518–6644658
RM6836	aa	9308941–9309108
RM3330	AA	11064158–11064302
RM3183	AA	12447059–12447198
RM1161	AA	13752128–13752207
RM3187	AA	20925773–20925914
RM4447	AA	22679594–22679732
RM1340	AA	23343196–23343361
RM3628	AA	23737084–23737180
RM7434	AA	23934836–23934978
RM162	AA	24035491–24035615
RM275	AA	24324733–24324821
RM5957	Aa	24521524–24521700
RM5314	Aa	24842804–24842954
RM5371	Aa	25825428–25825525
RM6395	Aa	25995534–25995643
RM3430	Aa	27432606–27432761
RM5509	Aa	27828211–27828465
RM3183	Aa	28469084–28469188
RM340	Aa	28599182–28599297
RM3509	aa	30970997–30971169

a A means allele originates from Y98149; a means allele originates from Hua-jing- xian74.

A contig map was constructed based on the reference sequence aligned by the *Sdt97* linked markers, and the mutant *Sdt97* locus was defined to a 118-kb interval within the PAC clone P0453H04 (Sequence ID:dbj|AP005453.1| length: 175,047 bp) (http://blast.ncbi.nlm.nih.gov/Blast.cgi).

**Figure 3:**
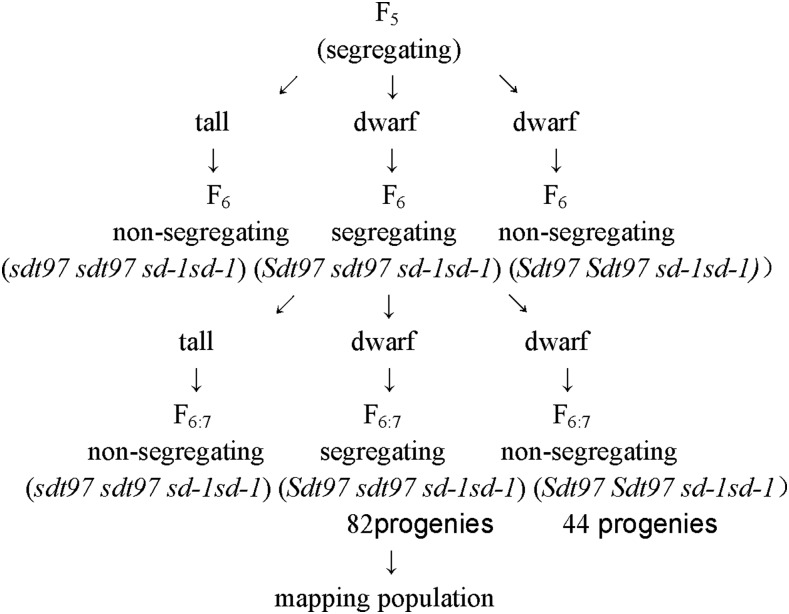
Plant height segregation mode of the F_6:7_ progenies derived from RHLs.

In the F_6:7_ progenies, 44 populations derived from homozygous dwarf F_6_ individuals, exhibited dwarf nonsegregation; 82 populations derived from heterozygote dwarf F_6_ individuals showed continued plant height segregation, and resulted in both tall and dwarf individuals (Figure 3). The ratio of the segregating F_6:7_ progenies to the nonsegregating dwarf F_6:7_ progenies is 1.86, which fits the expected Mendelian segregated ratio of 2:1 (χ^2^ = 0.081, *P* > 0.05). The results confirmed that there was only one dominant gene controlling the segregation of plant height in the RHL population.

**Figure 4:**
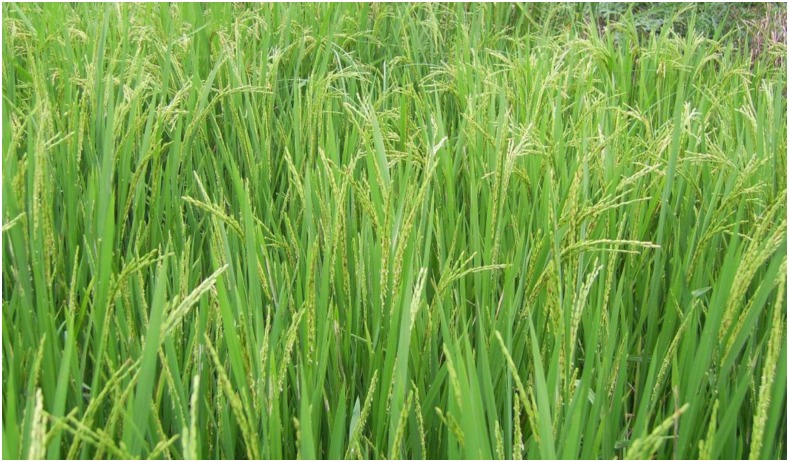
Semidwarf plants and dwarf plants in the RHL mapping population of segregated F_6:7_ progenies.

A large scale F_7_ RIL population, consisting of 10,000 F_7_ individuals, was constructed. Of these segregating F_7_ RHL plants, 2328 F_7_ tall recessive individuals were selected for the fine mapping of *Sdt97* using the RCA method (Figure 4, Tuinstra *et al.* 1997; Yamanaka *et al.* 2005; Tong *et al.* 2007). For genetic validation or progeny testing of the recessive F_7_ individuals, 2328 F_7:8_ RHL derived from these tall F_7_ recessive individuals were further planted in the field at the experimental stations in Sanya (18 × N, 109 × E), Hainan province, China. A total of 2312 of them, which passed progeny testing, was further used for the fine mapping of *Sdt97* in this study.

### Fine mapping of Sdt97

**Figure 5:**
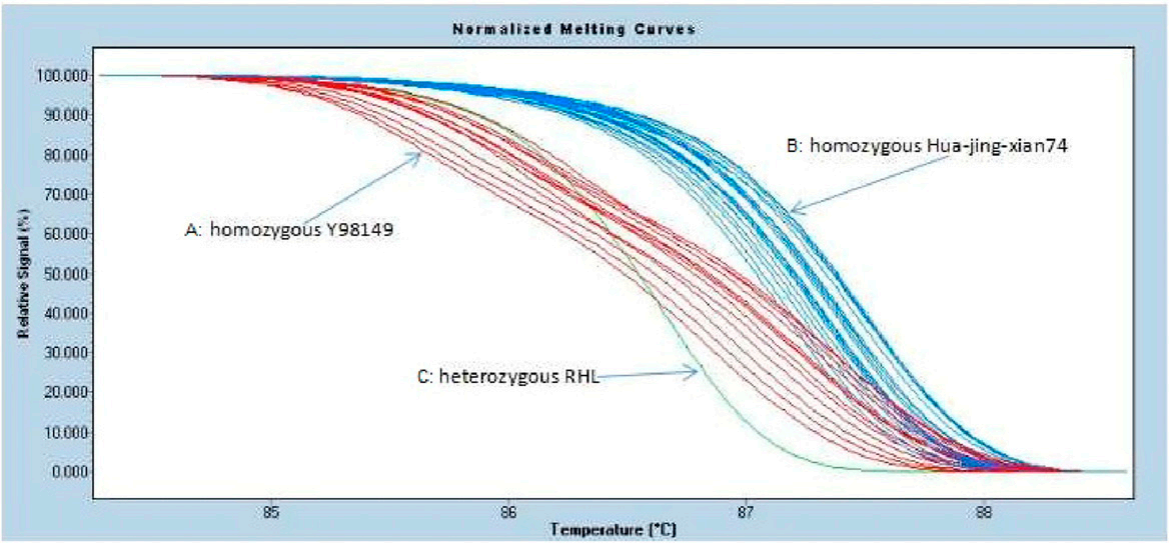
PCR products were amplified with SNP marker N16 from tall recessive individuals in the segregating F_6:7_ populations, and analyzed by high-resolution-melting (HRM). Three types of HRM curves were obtained: curve *A* represents homozygous Y98149, curve *B* represents homozygous Hua-jing-xian74, and curve *C* represents heterozygous RHL.

Upon further linkage analysis, one cleaved amplified polymorphic sequence (dCAPS) (*Bam*HI), and 16 SNP markers were developed by sequencing the PCR products amplified from Y98149 and Huajingxian74 in the target region (Supplemental Material, File S1). Depending on sequence variation, different SNP genotype of the recessive individuals in the F_6:7_ populations be differentiated (Figure 5).

A total of 2312 tall recessive individuals in the 86 F_6:7_ segregated population was used. By analyzing BSA and RCA, 33 distinct recombinants were identified in the target region. Using one dCAPS marker (*Bam*HI), and 16 new SNP markers developed in this research, the *Sdt97* gene was further mapped to the long arm of chromosome 6 at the interval of nearly 60 kb between STS markers N6 and SNP marker N16 within the PAC clone P0453H04 (Sequence ID: dbj|AP005453.1 |length: 175,047 bp). The sequence of primers N6 and N16, and their location on the PAC clone P0453H04, are as shown in Table 3 (see http://blast.ncbi.nlm.nih.gov/Blast.cgi).

**Table 3:** The sequences of primers N6 and N16, and their location on P0453H04

Marker	Forward Primer	Reverse Primer	Location on P0543H04
N6	GCCGGGGAGCTACTACCGAG	CACTGATTCAGCTCCCAAGGC	36129 bp–36327 bp
N16	GGGATAGATGCCTTCCATTGTT	CGAGTAGGAAGTGCCTCTAGCG	96547 bp–96761 bp

### Map-based cloning of the Sdt97 gene

Based on the available sequence annotation database (http://www.gramene.org/;http://rice.plantbiology.msu.edu/index.shtml), nine genes were predicted in the 60 kb target region of the cultivated rice genome. To identify the candidate gene of *Sdt97*, the genomic DNA sequence of all nine predicted genes in the 60 kb target region were sequenced. Based on the sequence analysis, nine genes could be categorized under three headings (Table 4).

**Table 4:** The information and sequence alignment of the genes in Y98148, Y98149 in the 60 kb target region

Gene	5′–3′	Putative Function	Sequence Alignment Result
*LOC_Os06g44040*	7096–10256	DOMON domain containing protein, expressed	No difference
*LOC_Os06g44050*	13373–16415	Methyladenine glycosylase, putative, expressed	One base mutation occurred in 5′-UTR region, and 342 bp nucleotide fragment deletion at the CDS region. The mutant base of mutant at the site of 277 bp upstream of initiation codon was C, which in wild type was G
*LOC_Os06g44060*	19082–23641	Phospholipase D.Active site motif family protein, expressed	No difference
*LOC_Os06g44070*	25659–27705	Retrotransposon protein, putative, unclassified	No difference
*LOC_Os06g44080*	29517–32089	Ubiquitin family protein, putative, expressed	No difference
*LOC_Os06g44090*	33739–34636	Hypothetical protein	No difference
*LOC_Os06g44100*	35862–37421	HLS, putative, expressed	No difference
*LOC_Os06g44120*	51318–51844	Retrotransposon protein, putative,Ty3-gypsy,subclass	Multi copy gene(2)
*LOC_Os06g44130*	52687–54379	Retrotransposon protein, putative, unclassified	Multi copy gene(3)

Note that the 342-bp deletion fragment in *LOC_Os06g44050* is observed between Y98149 (or Y98148) and Nipponbare.

There is no DNA sequence difference between the semidwarf mutant and the tall wild type; *LOC_Os06g44040*, *LOC_Os06g44060*, *LOC_Os06g44070*, *LOC_Os06g44080*, *LOC_Os06g44090*, and *LOC_Os06g440100* were of this type.

Multicopy genes exist in the rice genome; LOC_Os06g44120 and LOC_Os06g44130 were of this type. Two copies of LOC_Os06g44120 [LOC_Os06g44120; LOC_Os12g34630], and three copies of LOC_Os06g44130 [LOC_Os06g44130; LOC_Os03g19150; LOC_ Os10 g08300], are known to exist in the rice genome.

Sequence analysis of genomic DNA fragments of the gene from tall wild-type and the semidwarf mutant showed that a point mutation occurred in the 5′-UTR of *LOC_Os06g44050*, resulting in a transversion from G to C.

**Figure 6:**
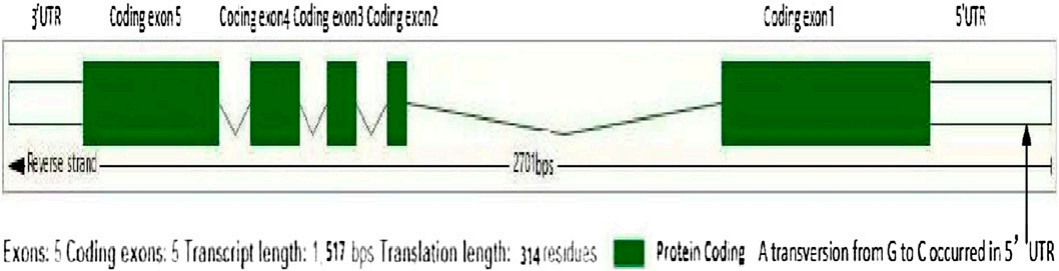
Schematic model of *Sdt97* gene.

**Figure 7:**
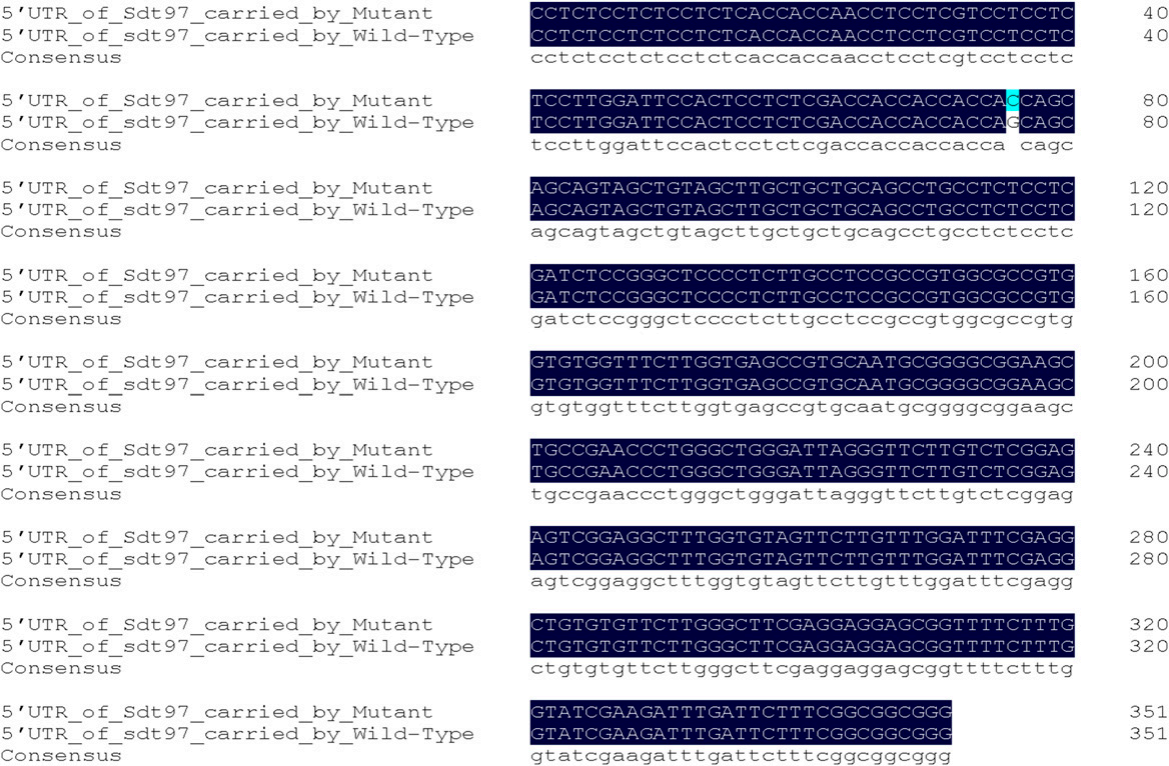
5′-UTR sequence alignment of *Sdt97* carried by mutant, and *sdt97* carried by wild type.

**Figure 8:**
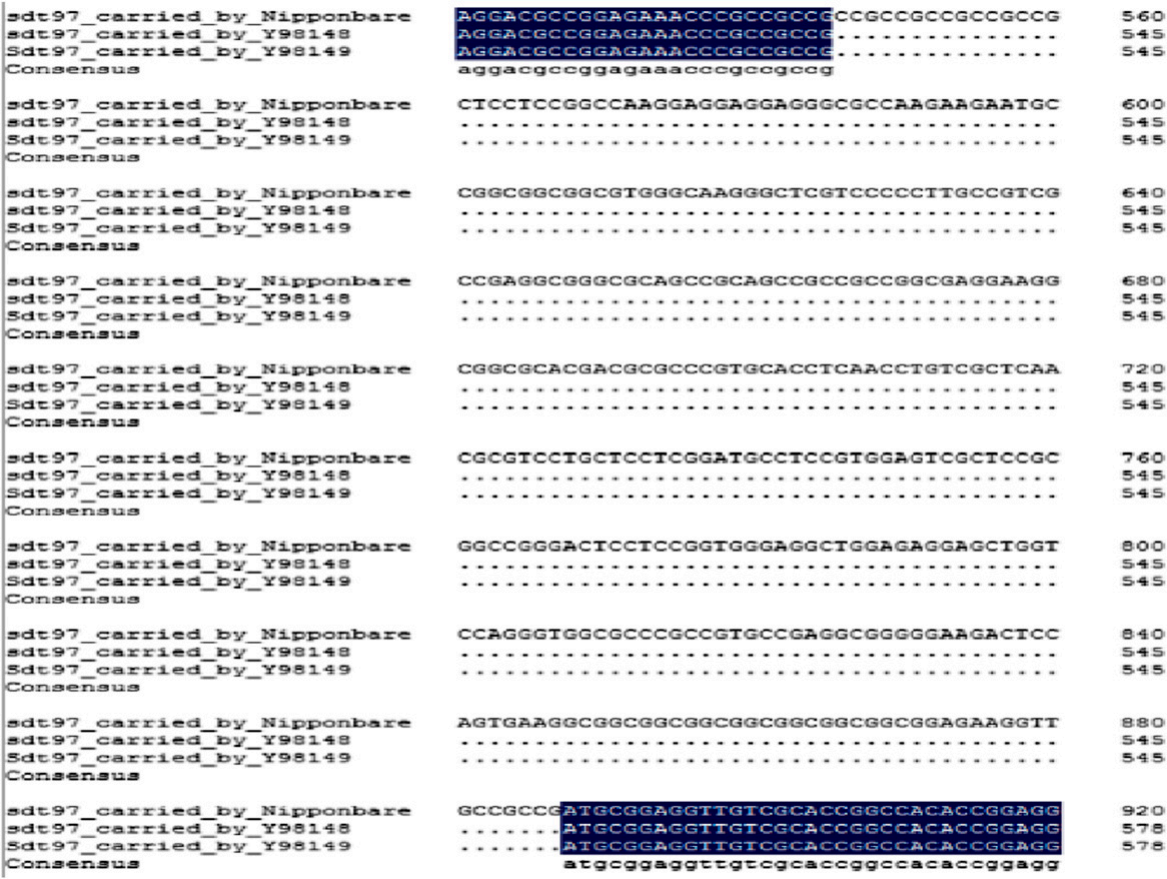
Fragment (342 bp) representing nucleotide deletion of *Sdt97* in mutant and *sdt97* in wild type.

The transversion took place at a site 277 bp upstream of the initiation codon in the 5′-UTR of exon 1 (Figure 6). The mutant base of *Sdt97* carried by Y98149 was C, while *sdt97* alleles of the same gene occupying the equivalent locus on homologous chromosomes carried by Y98148 had a G in this position (Figure 7). Compared with the genomic DNA sequence of Nipponbare, it was found that not only Y98149, but also Y98148, had a 342-bp deletion in the first coding exon in *LOC_ Os06g44 050*. The deletion occurred between 546 bp and 887 bp in the genomic sequence, or between 194 bp and 535 bp in the coding sequence (CDS) of *LOC_Os06g44050* (Figure 8). Thus, *LOC_Os06g44050* was finally identified as the candidate gene of *Sdt97*.

The genomic DNA and cDNA nucleotide sequences of *Sdt97* were 2701 bp and 1517 bp in length, respectively. *Sdt97* consisted of five exons and four introns, as follows: 5′-exon 1 [617 bp = 5′-UTR (352 bp) + coding exon 1 (265 bp)]–intron 1 (920 bp)–exon 2 (coding exon 2, 58 bp)–intron 2 (92 bp)–exon 3 (coding exon 3, 84 bp)–intron 3 (79 bp)–exon 4 (coding exon 4, 142 bp)–intron 4 (93 bp)–exon 5 [616 bp = coding exon 5 (396 bp) +3′-UTR (220bps)]–3′ (File S1).

A schematic gene model of *Sdt97* is displayed in Figure 6. The DNA and cDNA sequences of *Sdt97* and the deletion fragment are provided.

### Transversion from G to C in the 5′-UTR of Sdt97 alters the expression pattern

At different stages of growth and development of Y98149 and Y98148, qRT-PCR was applied to analyze the effect of the transversion from G to C in the 5′-UTR of *Sdt97* on its own gene expression. The results of qRT-PCR were as follows (Table 5):

**Table 5:** The relative quantification of *Sdt9*7 (carried by semidwarf mutant Y98149), and *sdt97 (*carried by tall wild-type Y98148) (NrelM/NrelWT) at different growth stages

Item	Seedling Stage	Tillering Stage	Elongation Stage	Milk Ripe Stage
ΔCt, *Sdt97*, carry in Y98149	−0.65	−2.42	−0.58	−0.97
ΔCt, *sdt97*, carry in Y98148	−0.77	−0.30	−2.64	−0.97
ΔΔCt = (ΔCt _S_*_dt97_*-ΔCt*_sdt97_*)	0.12	−2.12	2.06	0
N_relM_/N_relWT = 2_^-ΔΔCt^	0.92	4.37	0.24	1.00

At the seedling stage, the ΔΔCt = 0.12, the N_relM_/N_relWT = 2_^–ΔΔCt^ = 0.92, meaning that the gene expression of *Sdt97* in Y98149 was just the same level as that in Y98148.

At the tillering stage, the ΔΔCt = –2.12, the N_relM_/N_relWT = 2_^–ΔΔCt^ = 4.37, meaning that the gene expression of *Sdt97* in Y98149 was four times higher than that of *sdt97* in Y98148.

In contrast, at the elongation stage, the ΔΔCt = 2.06, the N_relM_/N_relWT = 2_^–ΔΔCt^ = 0.24, meaning that the gene expression of *sdt97* in Y98148 was four times higher than that of S*dt97* in Y98149.

At the milk ripe stage, the ΔΔCt = 0, the N_relM_/N_relWT = 2_^–ΔΔCt^ = 1.00, meaning that the gene expression of *sdt97* in Y98148 was just the same as, or just more than, *Sdt97* in Y98149.

The expression level of the gene *Sdt97* in the transgenic lines is very strong supporting evidence for the function of *sdt97*. In this study, qRT-PCR was applied at different growth stages to analyze the effect of the gene transfer of *Sdt97* on the expression pattern of this gene in transgenic T_1_ lines of F_4_. Table 6 provides qRT-PCR results.

**Table 6:** The relative quantification of the transgenic T_1_ lines of F_4_/the transgenic receptor, the tall wild-type, Y98148 (NrelF_4_/NrelWT) at different growth stages

Item	Seedling Stage	Tillering Stage	Elongation Stage	Milk Ripe Stage
ΔCt, *Sdt97*, carried in the transgenic T_1_ lines of F_4_	2.05	1.03	2.65	5.98
ΔCt, *sdt97*, carried in the tall wild-type Y98148	2.15	1.69	2.43	6.28
ΔΔCt = (ΔCt _S_*_dt97_*-ΔCt*_sdt97_*)	−0.1	-0.0.66	0.22	−0.3
N_relF4_/N_relWT = 2_^-ΔΔCt^	1.07	1.58	0.86	1.23

At the seedling stage, the expression level of the gene *Sdt97* in transgenic T_1_ lines of F_4_ was just the same as that in the transgenic receptor, the tall wild type, Y98148. At the tillering stage, the expression level of the gene *Sdt97* in the transgenic T_1_ lines of F_4_ increased, ΔΔCt = –0.66, the N_relF4_/N_relWT = 2_^–ΔΔCt^ = 1.58, *i.e.*, 1.58 times higher than that in the transgenic receptor, Y98148. At the elongation stage, in contrast, the expression level of the gene *Sdt97* in transgenic T_1_ lines of F_4_ decreased, ΔΔCt = 0.22, the N_relF4_/N_relWT = 2_^–ΔΔCt^ = 0.86, *i.e.*, lower than that in the transgenic receptor, Y98148. At the milk ripe stage, the expression level of the gene *Sdt97* in transgenic T_1_ lines of F_4_ was just the same as, or just slightly higher than that in the tall wild type Y98148.

The results showed that gene transfer of *Sdt97* induced obvious changes in the expression of this gene in transgenic T_1_ lines of F_4_, with a pattern similar to that in the semidwarfism mutant Y98149.

These results indicated that the G–C transversion that occurred in the 5′-UTR of *Sdt97* induced obvious changes in the expression pattern of the *Sdt97* gene carried in Y98149 and the transgenic lines, it altered the expression pattern of the gene *Sdt97* itself, and led to an obvious change in plant height performance of Y98149 and the transgenic lines.

### A transgenic Sdt97 line driven by a no-promoter version of the gateway vector (pGWB3) to tall wild type displays the semidwarf mutant phenotype

To highlight the effect of the point mutation in the 5′-UTR on the gene expression of *Sdt97* itself, a no-promoter Gateway vector, pGWB3 [(no promoter, C-GUS)(-R1-CmR-ccdB-R2-GUS-)] (Invitrogen), was used to carry out the complementarity test of *Sdt97* in this study.

**Figure 9:**
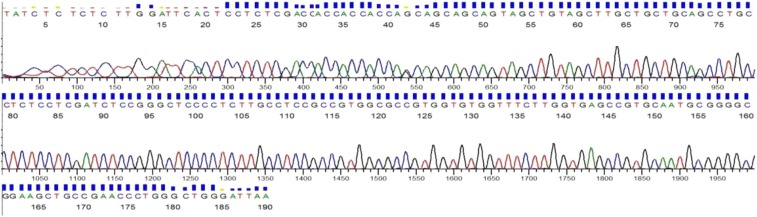
Determination of the transgenic plant genotype.

The genotypes of the transgenic plants were determined by sequencing PCR products. The transgenic acceptor lines with overlapping peaks of C and G at the nucleotide location of 124 bp in the DNA sequencing chromatogram can be easily and clearly detected (Figure 9).

**Figure 10:**
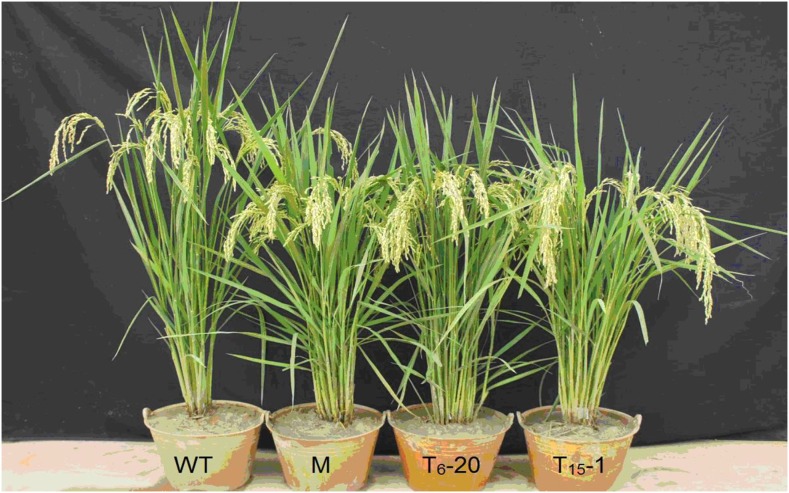
Height of the tall wild type (WT), the semidwarf mutant (M), and transgenic T_0_ for T_6_-20 and T_15_-1. From left to right: tall wild-type (WT, 100 cm), semidwarf mutant (M, 82 cm), transgenic T_0_ line for T_6_-20 (T_6_-20, 83 cm), and transgenic T_0_ line for T_15_-1 (T_15_-1, 85cm).

Forty-one independent transgenic T_0_ lines for T_6_, and 25 independent transgenic T_0_ lines for T_15_ were obtained. Most of the independent T_0_, T_1_ transgenic lines exhibited a semidwarf phenotype like that of the mutant (Y98149). Genetically, the positive transgenic T_0_, T_1_ plants were the same as that of the (Y98148/Y98149)F_1_,F_2_ progeny derived from crosses between Y98148 and Y98149. Because only pGWB3, which has no 35S promoter, was used in the transgenic clone construction of *Sdt97*, the CDS sequence of *Sdt97* in Y98149 was coincident with that of *sdt97* in Y98148. Therefore, the point mutation in the 5′-UTR region of *Sdt97* was responsible for restoration of the semidwarfism mutant phenotype in the transgenic lines (Figure 10).

### A Sdt97 transgenic line driven by the 35S promoter (tall wild type) displays a more pronounced dwarf phenotype than the mutant

**Figure 11:**
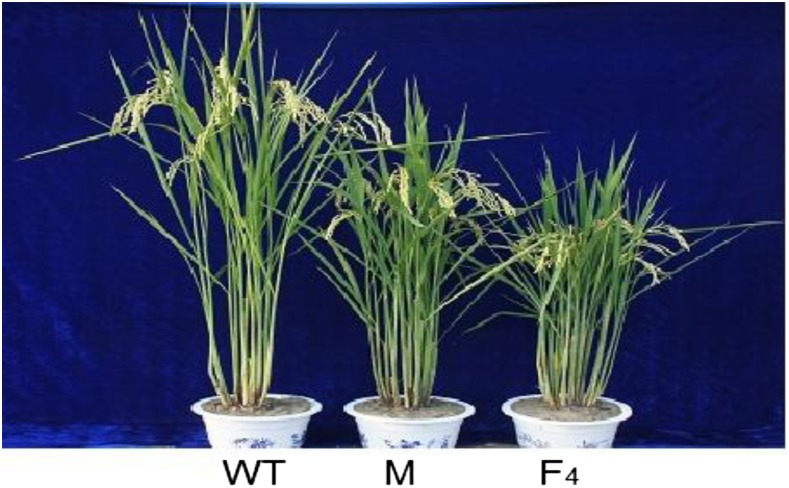
Height of the tall wild type (WT), semidwarf mutant (M), and transgenic T_1_ for F_4_. From left to right: tall wild type (WT, 103 cm), the semidwarf mutant (M, 85 cm), and the transgenic T_1_ line for F_4_ (F_4_, 68 cm).

In a complementarity test of *Sdt97*, we constructed transgenic plants. The mutant gene *Sdt97* driven by the 35S promoter (F_4_) was introduced into tall wild-type plants (Y98148) via *Agrobacterium*-mediated transformation. To confirm further that the mutated trait of semidwarfism was caused by a point mutation in the 5′-UTR of *Sdt97*, 14 independent transgenic T_1_ lines of the F_4_ generation were obtained. Most of the independent T_1_ transgenic lines exhibited a more pronounced dwarf phenotype than the semidwarf mutant (Y98149) (Figure 11). The results indicated that *Sdt97* overexpression in the genetic background of the tall wild type (Y98148) caused a more pronounced dwarf phenotype than the semidwarf mutant (Y98149).

## Discussion

### Sdt97, a new dwarf gene reported in rice

More than 70 dwarf and semidwarf genes have been identified in rice. These genes mediate important physiological and biochemical processes, and most of them are relevant to plant hormones. The semidwarf1 (*sd1*), designated as the ‘Green Revolution Gene’, affects the gibberellin (GA) biosynthesis pathway (Monna *et al.* 2002; Sasaki *et al.* 2002; Spielmeyer *et al.* 2002; Muangprom and Osborn 2004). The d35Tan-Ginbozu is caused by a defective early step in GA biosynthesis (Itoh *et al.* 2004). *OsGA3oxl* and *OsGA3ox2* encode proteins with 3b-hydroxylase activity (Itoh *et al.* 2001). The dwarf mutant genes *GID1*, *GID2*, dwarf mutant *d1*, and *slr1-1* in rice are defective in response to GA (Ueguchi-Tanaka *et al.* 2005; Hartweck *et al.*and Olszewski 2006; Gomi *et al.* 2004; Ueguchi-Tanaka *et al.* 2000; Ashikari *et al.* 1999; Ikeda *et al.* 2001). Brassinosteroids (BRs) are structurally defined as C27, C28, and C29 steroids with substitutions on the A- and B-rings and side chains (Fujioka and Sakurai 1997; Yokota 1997; Grove *et al.* 1979).The *brassinosteroid dependent 1* (*brd1*) is defective in BR-6-oxidase (Mori *et al.* 2002; Hong *et al.* 2002). *D2* encodes a novel cytochrome P450 classified as CYP90D, The D2/CYP90D2 is an enzyme catalyzing a step in the late BR biosynthesis pathway (Hong *et al.* 2003). The *brd2* gene is defective in the rice homolog of *Arabidopsis* DIMINUTO/DWARF1 (Hong *et al.* 2003). *D11* encodes a novel cytochrome P450 (CYP724B1), D11/CYP724B1 is involved in the BR biosynthesis network in rice (Tanabe *et al.* 2005). O*sdwarf4-1* has a weak dwarf phenotype of *OsDWARF4* (Sakamoto *et al.* 2006). Bai *et al.* (2007) identified OsBZR1-interacting proteins. *OsBRI1*, *d61-7*, *DLT*, and three MADS box proteins are defective in response to BRs in rice (Yamamuro *et al.* 2000; Morinaka *et al.* 2006; Tong *et al.* 2009; Duan *et al.* 2006; Lee *et al.* 2008). The strigolactones are rhizosphere-signaling molecules as well as a new class of plant hormones with an increasing number of biological functions (Ruyter-Spira *et al.* 2013). The *gsor23* encodes a key enzyme involved in the biosynthesis of strigolactones (SIs) (Wang *et al.* 2013). *D53* encodes a substrate of the SCFD3 ubiquitination complex and functions as a repressor of SL signaling (Jiang *et al.* 2013; Zhou *et al.* 2013). In 2012, a gain-of-function epi-allele (Epi-df) of rice fertilization-independent endosperm1 (*FIE1*) was identified by Zhang *et al.* (2012). *Epi-df* is the only dwarf mutant gene entirely unrelated to plant hormones reported in rice. It has no altered nucleotide sequence but is hypomethylated in the 59 region of *FIE1*, resulting in a dwarf stature (Zhang *et al.* 2012).

*Sdt97* was deduced to have resulted from a spontaneous mutation. In our previous research, *Sdt97* was mapped to the long arm of chromosome 6 at the interval between two STS markers, N6 and TX5, at a genetic distance of 0.2 cM and 0.8 cM, respectively. In a recent study, *Sdt97* was fine-mapped to a interval of nearly 60 kb within the PAC clone P0453H04, and *LOC_Os06g44050* was identified as the candidate gene of *Sdt97*. *Sdt97* is not allelic to the *sd-1* gene on chromosome 1 (Monna *et al.* 2002; Sasaki *et al.* 2002; Spielmeyer *et al.* 2002), *sd-t*, *sd-t2* on chromosome 4 (Li *et al.* 2001; Jiang *et al.* 2002 ; Zhao *et al.* 2005), *sd-g*, *sd-n* on chromosome 5 (Liang *et al.* 1994, 2004; Li *et al.* 2003), *D53* on chromosome 11 (Wei *et al.* 2006), or the dwarf gene *Dx* on chromosome 8 (Qin *et al.* 2008). Clearly, *Sdt97* is a novel semidwarf gene reported in rice (Tong *et al.* 2007).

The predicted bioinformatics results show that *LOC_Os06g44050* (the candidate gene of *Sdt97*) encodes a putative methyladenine glycosylase, a DNA repair enzyme (http://www.Gramene.org/; http://rice.plantbiology.msu.edu/index.shtml). It is located on the reverse DNA strand of chromosome 6 (Chromosome 6: 26,568, 419-26,571,461) (http://www.Gramene.org/; http://rice.plantbiology.msu.edu/index.shtml). Compared with *LOC_Os06g44050* carried by Nipponbare, the genomic DNA sequence of *Sdt97* carried by the semidwarf mutant (Y98149), and *sdt97* carried by tall wild type (Y98148), carry a 342-bp nucleotide deletion (Figure 8). The deletion occurred between 546 bp and 887 bp in the first exon, or between 194 bp and 535 bp in the CDS of *LOC_Os06g44050* carried by Nipponbare. The peptide encoded by *Sdt97* and *sdt97* is 114 amino acids shorter than the peptide encoded by *LOC_ Os06g44050*.

Until now, no phenotype, disease, or trait is known to be associated directly with *LOC_Os06g44050* (the candidate gene of *Sdt97*), and no phenotype, disease, or trait is associated with these gene variants in plants (www.gramene.org). Methyladenine glycosylase has been reported only in *Mycobacterium bovis* BCG (Liu *et al.* 2014). Therefore, further research into *Sdt97* will open new fields of research into rice semidwarfism, with emphasis on the regulation of *Sdt97* expression at the transcriptional level, and the relationship between point mutations in the 5′-UTR and *Sdt97* promoter, the relationship between methyladenine glycosylase and the semidwarf phenotype of the mutant, and the physiological and biochemical pathways of *Sdt97* at the molecular level.

### Sdt97 could be used as an elite cultivar to breed super-high-yielding hybrid rice in China

Dwarf resources are the foundation to realizing the ideal plant type in rice breeding (Zhu 1980). Since the 1960s, the discovery and identification of new dwarf gene resources in rice have gained huge attention. However, these genes are associated with traits such as severe dwarfism, floret sterility, or abnormal plant and grain development; therefore, most of them have not been used in crop improvement (Aquino and Jennings 1966; Kinoshita 1995;Tong *et al.* 2003). In recent years, new semidwarf genes nonallelic to *sd-1* have been identified (Liang *et al.* 1994, 2004; Li *et al.* 2001, 2003; Jiang *et al.* 2002; Zhao *et al.* 2005), but *sd-1* is still the primary semidwarf gene used in rice breeding (Kinoshita 1995). It is reported that about one-half of the stock from the IRRI collection is allelic to Dgwg (Gu & Zhu 1979). In southern China, 75% of the semidwarf gene found in varieties of economic importance is at the same locus as *sd-1* (Min *et al.* 1996).

Frequent usage of the *sd-1* gene may reduce genetic diversity and bring about genetic vulnerability to pests and diseases. According to reports, in Asia, the genetic distance of intersubspecies was nearly equal to that of intervariety in rice (Hong 1999), while the genetic distance of the improvement rice varieties, and their parents, was less than half of that of intersubspecies (Zhuang *et al.* 1997). It is therefore imperative to develop a new source in order to broaden the genetic basis of semidwarfism. The discovery and utilization of a dominant semidwarf gene may be of great significance, not only in genetic theory but also in rice breeding practice (Tong *et al.* 2001, 2003).

The semidwarf mutant genes reported here are just the ideal gene resources of the dominant semidwarf, and could provide a power genetic tool not only to resolve the problem of excessive height in the inter-subspecific F_1_ hybrid, but also to breed an ideal type of ‘super-high-yielding rice’. In our previous research, the semidwarf mutant gene *Sdt97* and the photoperiod-sensitive genic male sterile gene *pms3* were combined, and a series of semidwarf PSGMS rice was bred successfully, which is now available for a two-line hybrid rice breeding program (Tong *et al.* 2007).

## Supplementary Material

Supplemental Material
