# Supplementary material for: Sdt97: A Point Mutation in the 5′ Untranslated Region Confers Semidwarfism in Rice
Source: G3 (Bethesda). 2016 Apr 28;6(6):1491–502. doi: 10.1534/g3.116.028720 (PMC4889646; doi:10.1534/g3.116.028720)
Supplement: Supplemental Material [file supp_6_6_1491__index.html]

Sdt97: A Point Mutation in the 5′ Untranslated Region Confers Semidwarfism in Rice — Supplemental Material 

# *Sdt97*: A Point Mutation in the 5′ Untranslated Region Confers Semidwarfism in Rice

## Supplemental Material for Tong *et al.*, 2016

**Files in this Data Supplement:**

- File S1 - Supplementary material (.zip, 14 KB)
