# Supplementary material for: Sdt97: A Point Mutation in the 5′ Untranslated Region Confers Semidwarfism in Rice
Source: G3 (Bethesda). 2016 Apr 28;6(6):1491–502. doi: 10.1534/g3.116.028720 (PMC4889646; doi:10.1534/g3.116.028720)
Supplement: Supplemental Material [file supp_g3.116.028720_FileS1.zip › 16 Pairs of PCR Primers for HRM-SNP Genotyping in the Target Region.doc]

**16对HRM-SNP引物序列资料。**

**01,HRM-N8-1F：CTAGGGACTGCTTATGGCGC，**

**HRM-N8-1R：CCGTTGGTGTTAGAGAAATCCA。**

**02,HRM-N15-1F：GAGAACGCAACGATGCAATG，**

**HRM-N15-1R ：GCTTGATTGGCGTTTACTACTACC。**

**03,HRM-N16-1F：GGGATAGATGCCTTCCATTGTT，**

**HRM-N16-1R：CGAGTAGGAAGTGCCTCTAGCG。**

**04,HRM-N22-1F：CAGCTCAGCTTGGTCACTGC，**

**HRM-N22-1R：GCCAGTGTCCAAGTAATTTTGC。**

**05,HRM-N27-2F：GCTCCTCCACCGCATCATC，**

**HRM-N27-2R：TGATCCGAGATCTTGCGGTG。**

**06,HRM-2Ag-1F：GCAGATACTCCCAGATCACCCT，**

**HRM-2Ag-1R：CTCCTTCAAATCCACGCACG；**

**07,HRM-2Ag-2F：CATAGGTCGCCGCTCTTCAG，**

**HRM-2Ag-2R：GAGGAGATCACCAGCAACCG；**

**08,HRM-2Ag-3F：CGATGAAGTAGGAGGCGACG，**

**HRM-2Ag-3R：GACCTTCTTCTGGACGCTCTC。**

**09,HRM-7Ag-1F：GGCTGTATGTTCCGAAGCTGAC，**

**HRM-7Ag-1R：AAGACCGGACCTGCATTGG。**

**10,HRM-11Ag-1F：GCTCCGTCGTCGGTTCCTG，**

**HRM-11Ag-1R：GCAGGGGACCAGGATAGGATAG。**

**11,HRM-17Ag-1F：GCGGCGTAGAGGAAGAGGTAG，**

**HRM-17Ag-1R：CTCTTCCTCGTCTTCGCCAT；**

**12,HRM-17Ag-2F：ACACCAGCAGCATCGCCG，**

**HRM-17Ag-2R：AAGCTGGTGCACGGCGA。**

**13,HRM-20Ag-1F：CTGATCGCCATAGAAGGATGC，**

**HRM-20Ag-1R：CAGATCATCATTTGAAGCACTGTC；**

**14,HRM-20Ag-2F：GACAGTGCTTCAAATGATGATCTG，**

**HRM-20Ag-2R：CTGAATTTGGAACGTGTACGGC。**

**15,HRM-24Ag-1F：GTGTGTAAACTCGGACCCAAA，**

**HRM-24Ag-1R：ATTGTGGGCCTCGCATGTCT；**

**16,HRM-24Ag-2F：AGTACAATAACACGGCGGAGG，**

**HRM-24Ag-2R：****GGTGGAGTCCTCCTTGAGCG。**
